# Insulin analogues dosing and costs - comparing real-life daily doses of insulin detemir and insulin glargine in type 2 diabetes patients

**DOI:** 10.1186/1472-6823-12-21

**Published:** 2012-09-25

**Authors:** Marie Jakobsen, Mette Dalsgaard, Morten Hørmann, Daniél Vega Møller

**Affiliations:** 1 COWI A/S, Parallelvej 2, Kongens Lyngby, DK-2800, Denmark; 2 Novo Nordisk Scandinavia AB, Arne Jacobsens Allé 17, 9, København, DK-2300, Denmark

**Keywords:** Insulin detemir, Insulin glargine, Type 2 diabetes, Dose, Health care costs

## Abstract

**Background:**

The uncertainties regarding dose similarities between basal long-acting insulin analogues remain. Recent real-world studies indicate dose similarities between insulin detemir and insulin glargine, but further studies are still warranted.

The aim of this study was to compare real-life daily doses of insulin detemir and insulin glargine in type 2 diabetes patients when administered once daily.

**Methods:**

We analysed 536 patient cases from general practice (63%) and endocrinological outpatient clinics (37%). A self-administered questionnaire completed by the treating physician was used to obtain data on patient characteristics (gender, age, weight, height, latest HbA_1c_-value), daily doses, administration of and number of years treated with insulin detemir and insulin glargine, concomitant insulin use and use of non-insulin anti-diabetic medication. Both bivariate analyses and multivariate regression analyses were applied to examine whether there were differences in the daily doses of insulin detemir and insulin glargine.

**Results:**

There was no significant difference in the mean daily doses of insulin detemir (0.414 U/kg) and insulin glargine (0.416 U/kg) (p = 0.4341). In multivariate regression analyses, age and BMI had a significant influence on daily insulin dose with the dose increasing 0.003 U/kg (p = 0.0375) and 0.008 U/kg (p = 0.0003) with every 1 increment in age and BMI, respectively.

**Conclusions:**

Dose similarities between insulin detemir and insulin glargine were seen in type 2 diabetes patients when administered once daily. Thus, the use of insulin detemir and insulin glargine is not associated with different medical costs if the price and treating algorithm are similar.

## Background

Type 2 diabetes (T2D) is a chronic and potentially disabling disease that affects around 350 million people worldwide [1]. In Denmark, around 230,000 people have been diagnosed with T2D corresponding to 8% of the population aged 40+ years [2].

Glycemic control is important for the prevention of diabetes-related complications in T2D patients, e.g. heart disease, stroke, high blood pressure, blindness, kidney disease, neuropathy and amputations [3]. To obtain glycemic control (e.g. HbA_1c_<7.5%), T2D patients benefit from measures to improve insulin sensitivity such as diet and exercise management [4]. When these measures fail, glycaemic goals can often be achieved with oral anti-diabetic medication and/or injectable GLP-1 analogues. As the disease progresses, the majority of patients will require insulin to maintain HbA_1c_ at desired target levels. Insulin can be used concomitant to oral anti-diabetic medication/GLP-1 analogues and as a part of either a basal only or a basal-bolus regimen. Currently available basal insulin preparations include the two long-acting insulin analogues - insulin detemir (DET) [Levemir; Novo Nordisk, Denmark] and insulin glargine (GLAR) [Lantus; Sanofi-Aventis, USA] - as well as the intermediate-acting human insulin, neutral protamine Hagedorn (NPH) insulin [5]. Compared to intermediate-acting insulin (NPH), long-acting insulin analogues offer a prolonged duration of action and reduced risk of hypoglycaemic events, especially nocturnal events [6-11].

Other studies - including both clinical trials [12-15] and real-world studies [16];[17] - have found that the use of DET and GLAR in T2D patients results in comparable HbA_1c_ improvements and a similar low risk of hypoglycaemia versus NPH, whereas DET is associated with significantly less weight gain than GLAR [12-18].

However, uncertainties with regard to dose similarities between DET and GLAR remain. The attempt to compare daily doses of DET and GLAR has been complicated by different treatment algorithms where DET is dosed once or twice daily whereas GLAR is dosed only once daily. Thus, existing clinical trials comparing DET and GLAR provide inconsistent results in terms of dose-related findings. Some studies have concluded that the daily DET dose is on average higher than the daily GLAR dose [12];[15] whereas others find no significant differences [14]. Recent real-world studies indicate dose similarities between DET and GLAR [19-21].

The aim of this study was - in routine clinical settings in Denmark - to compare daily doses of DET and GLAR in T2D patients when administered once daily.

## Methods

### Data collection

Data was collected by a self-administered questionnaire to general practitioners (GPs) and specialists. The questionnaire included information on patient characteristics (gender, age, weight, height, latest HbA_1c_-value), use of insulin and non-insulin anti-diabetic medication. In total, 490 GP offices were contacted by letter (72), telephone (146) or online (272), and 86 endocrinological outpatient clinics were contacted by telephone. The GPs were asked to fill in a questionnaire for each of their T2D patients treated with either DET or GLAR based on information registered in their computer system. Data from GPs were collected from 6 December 2010 to 4 February 2011. The specialists were asked to fill in a questionnaire for each of their T2D patients treated with DET or GLAR whom they were in contact with from 29 November to 10 December 2010. In total, 79 GP offices and 25 endocrinological outpatient clinics participated in the study.

**Figure 1:**
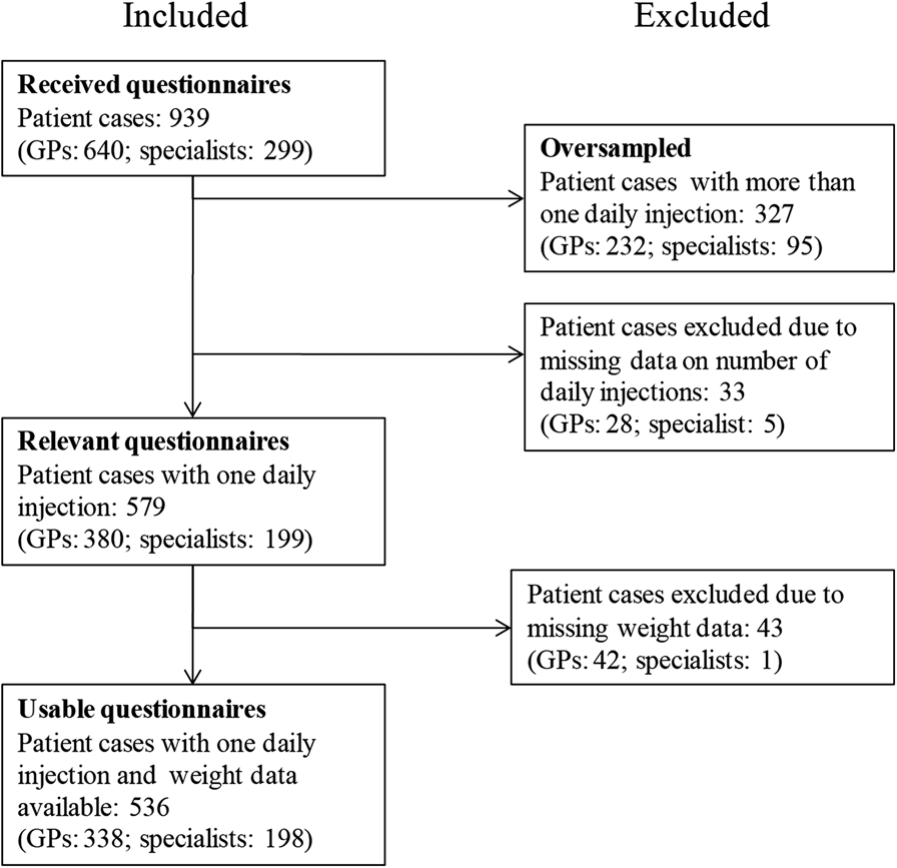
Patient case included in the study.

As illustrated in Figure 1, the participating GP offices and specialists returned 640 questionnaires and 299 questionnaires, respectively, providing data on 939 patient cases in total. Among the 939 patient cases, 360 were found ineligible because DET or GLAR was administered more than once daily or data on number of daily injections was not available. Furthermore, 43 patient cases were excluded because of missing body weight data. In total, 536 patient cases were included in the analyses.

### Statistical methods

Analyses were performed using the statistical package SAS 9.2 for Windows (SAS Institute, Cary, NC, USA). Both bivariate analyses and multivariate linear regression analyses were applied to examine possible differences in the daily doses of DET and GLAR. The following background variables were included as covariates in the multivariate regression analyses: gender, age, height, BMI, region, HbA_1c_-value, prescriber (GP or specialist), concomitant insulin use, non-insulin anti-diabetic medication, number of years treated with DET or GLAR and number of years on anti-diabetic medication. Two models were estimated - a small and a larger model in terms of number of covariates. Patient cases with missing values were excluded from the analyses. All statistical tests were performed at 5% significance level.

### Ethics

Approval from an ethics committee was not required by Danish law since the study did not involve collection of or research on biological material. Furthermore, as no personal data defined as any information relating to an identified or identifiable patient was obtained in the questionnaires to GPs and specialists (e.g. CPR number (civil identification number) or other personal data which could refer to a single patient), no informed consent was necessary by Danish law. A waiver for ethics approval has been obtained.

## Results

### Bivariate analysis

There was no significant difference in the mean daily doses of DET and GLAR (see Table 1). The mean daily dose of DET (n = 251) was 0.414 U/kg and the mean daily dose of GLAR (n = 285) was 0.416 U/kg (p = 0.4341).

**Table 1:** Mean daily dose of long-acting insulin analogue DET and GLAR (U/kg)

	**Detemir**	**Glargine**	**P-value **(**χ^2^**-test)****
N	251	285	
Mean daily dose (U/kg)	0.414	0.416	0.4341

Background characteristics of the study population are shown in Table 2. No significant differences between DET and GLAR patients were found with respect to gender, age, weight, height, BMI, latest HbA_1c_-value and use of non-insulin anti-diabetic medication. The percentage of DET patients was larger among GPs (71%) than among specialists (56%) (p = 0.0002). Furthermore, significant or borderline significant differences were found in concomitant insulin use with more DET patients treated with Actrapid and more GLAR patients treated with Insuman Rapid. However, the overall percentage of T2D patients in the study population treated with these products was very small, and it is unclear whether such differences exist for T2D patients in general.

**Table 2:** T2D patient characteristics by basal long-acting insulin analogue DET and GLAR

	**Detemir**		**Glargine**		**P-value**
	**N**	**(%)**	**N**	**(%)**	**(**χ^2^**-test)**
Total	251	47%	285	53%	
Patient Characteristics					
Female	101	40%	130	46%	0.2099
Male	150	60%	155	54%	
Mean age	61		60		0.7123
<50 years	49	20%	63	22%	0.4629
≥50 years and <60 years	96	38%	103	36%	0.6145
≥65 years	106	42%	119	42%	0.9112
Mean weight (kilos)	86		87		0.2044
Mea height (cm)	172		172		0.4055
Mean BMI (kilos/m^2^)	29.1		29.8		0.3587
HbA1c value (%)	8.0 (63.9 mmol/mol)		8.2 (66.1 mmol/mol)		0.5602
Mean number of years on antdiabetic medication	11		10		0.068
Mean number of years on Determir/Glargine	2.1		2.2		0.2672
Geography					
North Denmark Region	22	9%	26	9%	0.8849
Central Denmark Region	63	25%	32	11%	0.0000
Southern Denmark Region	66	26%	85	30%	0.3647
Region Sealand	43	17%	44	15%	0.5959
Capital region of Denmark	57	23%	98	34%	0.0029
Prescriber					
GP	179	71%	159	56%	0.0002
Specialist	72	29%	126	44%	
Concomitant insulin use					
At least one product	110	44%	114	40%	0.3703
NovoRapid	79	31%	82	29%	0.4959
Actrapid	16	6%	4	1%	0.0024
Insuman Rapid	0	0%	4	1%	0.0596
Aprida	0	0%	3	1%	0.1031
Other insulin	18	7%	22	8%	0.8096
Non-insulin antdiabetic medication					
At least one product	159	63%	188	66%	0.5267
Biguanides	146	58%	173	61%	0.5509
Sulfonomides	15	6%	16	6%	0.8578
Glitazoner (TZD) and combinations	0	0%	0	0%	
DPP-IV inhibitors and combinations	8	3%	4	1%	0.1637
Victoza	26	10%	41	14%	0.1595
Byetta	0	0%	1	0%	0.3476

### Multivariate regression analysis

The results of the multivariate regression analyses are reported in Table 3. In both models, type of long-acting insulin analogue used was not significant, i.e. the daily doses of DET and GLAR did not differ significantly between T2D patients when administered once daily and when taking into account other factors that may influence insulin utilisation.

**Table 3:** Multivariate regression models of daily dose of long-acting insulin analogue DET and GLAR (U/kg)

	**Model 1 (N-485, Adj R**^**2**^ **= 0.7636)**		**Model 2 (N-458, Adj R**^**2**^ **= 0.7699)**	
	**Coefficient**	**P-value (t-test)**	**Coefficient**	**P-value (t-test)**
Long-acting insulin analogue used (DET = 0, GLAR = 1)	−0.0010	0.9609	0.0009	0.9687
Patient characteristics				
Gender			0.0522	0.0884
Age	0.0026	<.0001	0.0019	0.0375*
Height			−0.0031	0.0905
BMI	0.0082	<.0001	0.0021	0.0003*
HbA1c value			0.0081	0.6428
Number of years on antidiabetic modification			0.0014	0.7045
Number of years on DET or GLAR			0.0067	0.0858
Geography				
North Denmark Region			0.5190	0.1194
Central Denmark Region			0.6017	0.0705
Southern Denmark Region			0.5495	0.0986
Region Sealand			0.4502	0.1766
Capital region of Denmark			0.5674	0.0860
Prescriber (GP = ,specialist)			0.0272	0.1806
Concomitant insulin use (none = 0, at least one other product = 1)			0.0259	0.3303
Non-insulin antidiabetic medication (none = 0, at least one other product = 1)	0.0295	0.2177	0.0278	0.0210*

Age and BMI, on the other hand, had a significant influence on the daily insulin dose with the dose increasing 0.003 U/kg (p = 0.0375) and 0.008 U/kg (p = 0.0003) with every 1 increment in age and BMI, respectively. The correlation between BMI and insulin dose is well-established. Obese T2D patients require larger doses of insulin to achieve metabolic control than lean T2D patients as they are more insulin resistant [4].

No other covariates included in the models showed robust statistical correlation with daily insulin dose. Neither concomitant insulin use nor use of non-insulin anti-diabetic medication was significant. This is probably a consequence of effects pulling in different directions. On the one hand, concomitant insulin use and use of non-insulin anti-diabetic medication are correlated with disease severity and thus daily dose of DET or GLAR. On the other hand, these products may act as substitutes for long-acting insulin analogues and thus reduce the daily dose of DET and GLAR.

## Discussion

This study contributes with real-life evidence of dose similarities between DET and GLAR in T2D patients in Denmark. No differences in daily doses of DET and GLAR were identified when administered once daily and when taking into account different factors that may influence insulin utilisation. Thus, use of DET and GLAR is not associated with different medical costs if the price and treatment algorithm are similar.

The results of this study are consistent with recent real-world studies from Europe and the US, which also did not find any significant dose differences between DET and GLAR in T2D patients [19-21].

The results of this study are also in line with available data from the Danish Medicines Agency on redeemed prescriptions to individuals in 2008 and 2009, which do not indicate differences in daily doses of DET and GLAR (unpublished observations).

The evidence from clinical trials comparing DET and GLAR is inconsistent in terms of dose-related findings. This study is consistent with the results of a clinical trial including 385 American T2D patients in a basal-bolus regimen, which did not find significant dose differences between DET and GLAR [14]. On the other hand, our results conflict with the results of a clinical trial including 582 European T2D patients in a basal regimen [15] and a clinical trial including 319 European and American T2D patients in a basal-bolus regimen [12]. Both studies found that the mean daily dose of DET was higher than the mean daily dose of GLAR. However, these differences were mainly due to differences in treatment algorithms with more DET patients on twice daily dosing compared to GLAR patients.

Clinical trials are often considered the gold standard, but they do not necessarily reflect clinical practice, and furthermore, clinical practice may differ between countries. Thus, real-world studies such as the present study play an important role in terms of providing evidence of actual insulin utilisation.

A major strength of this study is that data was collected directly from the treating physician giving precise information on prescribed insulin dose in units per day. The daily dose prescribed by the treating physician is not necessarily the same as the dose actually used by the patient. However, it does not seem likely that there are differences between DET and GLAR patients with regard to compliance, and therefore this should not influence the main conclusion of no significant dose differences between DET and GLAR. Overall, the risk of information bias is considered low in this study compared to other real-world studies where the daily insulin dose is calculated based on dispensing data [19-21].

Dose comparisons were strictly performed for the same treatment algorithm (once daily dosing of DET or GLAR) in accordance with the aim of the study (as it otherwise would include off-label use of GLAR). Patients with two or more daily injections − of which the majority was DET patients − were excluded in the analyses presented here which could be regarded as a limitation towards the ability to conclude on cost differences. However, multivariate regression analyses were performed with these patients included. The analyses confirmed the expected positive correlation between number of daily injections and daily insulin dose as well as dose similarities between DET and GLAR when administered twice daily (data not shown). The overall conclusion remained unchanged, i.e. no differences in daily doses of DET and GLAR were identified when taking into account different factors that may influence insulin utilisation, including treatment algorithm.

A limitation of this study is the sensitivity to selection bias as the participating GP offices and specialists were not selected randomly. However, the study population is large and diverse which makes it highly probable that the main conclusion of no significant dose differences between DET and GLAR in a once daily regime is representative for all T2D patients in Denmark.

Another limitation concerns the possibility of residual confounding. The existence of co-morbidities in T2D patients may influence insulin utilisation, but was not included in the multivariate regression models as data was not available. However, it is not likely that this would have changed the main conclusion of no significant dose differences between DET and GLAR as there are no compelling reasons to assume that co-morbidities differ between T2D patients treated with DET and GLAR.

## Conclusions

Based on this study, we conclude that the real-life daily doses of DET and GLAR in Danish T2D patients are similar when administered once daily. Thus, neither DET nor GLAR should be preferred over the other on economic grounds with reference to possible dose differences as long as the price and treating algorithm are similar.

## Abbreviations

DET: insulin detemir; GLAR: insulin glargine; NPH: neutral protamine Hagedorn insulin; T2D: type 2 diabetes; HbA_1c_: glycated hemoglobin (average plasma glucose concentration over prolonged periods of time); BMI: body mass index; GP: general practitioner; U: unit; Kg: kilo; Cm: centimetre; M: metre.

## Competing interests

Insulin detemir is produced and marketed by Novo Nordisk. COWI has received funding from Novo Nordisk Scandinavia to conduct the analyses and to draft the manuscript. Daniél Vega Møller is an employee of Novo Nordisk Scandinavia.

## Authors’ contributions

MJ: conception and design of the study; analysis and interpretation of data; drafting the manuscript; MD and MH: conception and design of the study; analysis and interpretation of data; DVM: conception and design of the study; acquisition and interpretation of data. All authors read and approved the final manuscript.

## Pre-publication history

The pre-publication history for this paper can be accessed here:

http://www.biomedcentral.com/1472-6823/12/21/prepub
